# Targeting cellular pathways in glioblastoma multiforme

**DOI:** 10.1038/sigtrans.2017.40

**Published:** 2017-09-29

**Authors:** Joshua R D Pearson, Tarik Regad

**Affiliations:** 1 The John van Geest Cancer Research Centre, School of Science and Technology, Nottingham Trent University, Clifton Lane, Nottingham, UK

## Abstract

Glioblastoma multiforme (GBM) is a debilitating disease that is associated with poor prognosis, short median patient survival and a very limited response to therapies. GBM has a very complex pathogenesis that involves mutations and alterations of several key cellular pathways that are involved in cell proliferation, survival, migration and angiogenesis. Therefore, efforts that are directed toward better understanding of GBM pathogenesis are essential to the development of efficient therapies that provide hope and extent patient survival. In this review, we outline the alterations commonly associated with GBM pathogenesis and summarize therapeutic strategies that are aimed at targeting aberrant cellular pathways in GBM.

## Introduction

Glioblastoma multiforme (GBM, WHO grade 4) is the most frequently occurring malignant central nervous system tumor with a global incidence of 0.59–3.69 per 100 000.^1^ It is by far the most common and malignant of all glial tumors, and is associated with poor prognosis with a median patient survival of 12–15 months from diagnosis.^2,3^ Unfortunately, only around 3–5% of patients survive for a period of 3 years or more.^4,5^ Although GBM affect primarily the cerebral hemispheres of adult brains, they are much less common in children, where they affect specifically the brainstem region. GBMs are classified as either primary or secondary, roughly 90% of cases are primary and occur *de novo* in elderly patients. Secondary cases progress from lower grade astrocytomas and are more prevalent in younger patients. Primary and secondary GBMs have differing genetic profiles with *IDH1* mutations being evident in secondary GBM and not primary.^6^ Common genetic alterations are associated with a loss of heterozygosity (LOH) of the chromosome arm 10q, that occur in 60–90% of GBM cases.^7,8^ Other alterations and deletions that affect the *p53* gene could be as high as 85.3–87%.^9,10^ *P53* alterations are more common in secondary GBMs than primary GBM tumors.^6^ Mutations in the epidermal growth factor receptor (EGFR) and in the platelet-derived growth factor receptor (PDGFR) are also associated with GBM pathogenesis and account for 40–57%^(refs. 9–11)^ and 60%^(ref. 12)^ subsequently. Other mutations target the gene of the mouse double minute homolog 2 (*MDM2*)* *(10–15%)^13^ and the phosphatase and tensin homolog (*PTEN*) gene (20–34%).^14,15^ Interestingly, genomic analyses performed by the Cancer Genome Atlas Research Network has revealed further alterations in key signaling pathways that contribute to the pathology of the disease. The RTK/Ras/PI3K signaling pathway was found to be altered in 86–89.6% and the pRB signaling pathway was found to be affected in 77–78.9% of GBM cases studied.^9,10^ It is important to add, that mutations encountered in GBM may not affect one single cellular pathway but may be the result of alterations in several of the pathways mentioned above. This adds further complexity to our understanding of GBM pathogenesis and results in additional complexity for the development of GBM therapies.

Regrettably, patients who are affected by GBM have a poor prognosis and existing therapies do not appear to be very efficient against GBM. The current gold standard for the treatment of GBM is palliative and includes surgery, adjuvant radiotherapy and temozolomide (TMZ) chemotherapy. Despite multimodal aggressive therapy, GBM is uniformly fatal with survival over 3 years being considered long-term.^16^ Due to the poor survival rate of GBM patients, it is imperative that novel avenues for therapy are explored in order to improve patient prognosis and eventually develop a cure to this fatal disease.

## Receptor tyrosine kinase pathways

### The tyrosine kinase receptors

**Figure 1:**
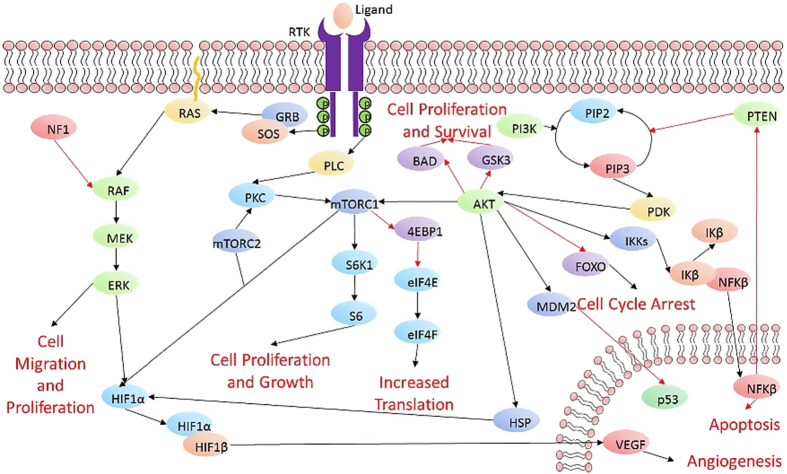
Schematic representation of RTK activation and the resultant downstream signaling. Black arrows indicate activation whereas red arrows indicate inhibition.

Receptor tyrosine kinases (RTKs) are a family of cell surface receptors, which act as receptors for growth factors, hormones, cytokines, neurotrophic factors and other extracellular signaling molecules. Upon activation by ligands, RTKs signal through two major downstream pathways Ras/MAPK/ERK and Ras/PI3K/AKT ^17^ (Figure 1). These pathways are involved in the regulation of cell proliferation, survival, differentiation and angiogenesis. In this review, we focus on six tyrosine kinase receptors; the epidermal growth factor receptor (EGFR), the vascular endothelial growth factor receptor (VEGFR), the platelet-derived growth factor receptor (PDGFR), the hepatocyte growth factor receptor (HGFR/c-MET), the fibroblast growth factor receptor (FGFR) and the insulin-like growth factor 1 receptor (IGF-1R).

Tyrosine kinase receptors share a similar structure that is composed of an extracellular ligand-binding domain, a hydrophobic transmembrane domain and an intracellular tyrosine kinase domain. They are activated by ligand binding which results in receptor dimerization and autophosphorylation of the tyrosine kinase domain. This event results in activation of two main downstream signaling pathways: Ras/MAPK/ERK and Ras/PI3K/AKT.^18–24^ Due to the ability of these receptors to activate downstream signaling pathways that are involved in proliferation, invasiveness, survival and angiogenesis, RTKs and their ligands are promising therapeutic targets for the treatment of GBM (Figure 1).

### EGFR

EGFR belongs to a family of four tyrosine kinases that encompasses ErbB1 (EGFR, HER1), ErbB2 (Her-2, Neu), ErbB3 (Her-3) and ErbB4 (Her-4). Amplifications and mutations in *EGFR (HER1)* were detected in 45–57% of GBM cases studied^9,10^ indicating a causal role in the pathogenesis of GBM. EGFRs induce proliferation and have been implied in glioblastoma pathogenesis and resistance to treatment.^25^ Interestingly EGFR is not the only member of this family that is mutated in GBM. ErbB2/HER-2 mutation was also detected in 8–41% of GBM cases.^9,26^ A truncated mutant EGFR variant III (EGFRvIII) is frequently expressed in glioblastoma multiforme and is constitutively activated in a ligand independent manner, resulting in cell proliferation and survival. Despite the growth enhancing properties of the EGFRvIII, its expression has been linked to increased overall survival in patients.^27,28^ This could be explained by the fact that EGFRvIII is a neoantigen and this may result in the elicitation of an immune response.

### VEGFR

VEGF is a potent angiogenic protein that is known to increase vascular permeability. Although VEGF has a role in normal tissues, malignant transformation has been shown to induce VEGF expression.^29^ Under hypoxic conditions, the hypoxia inducible transcription factors (HIF1α and HIF1β) translocate to the nucleus and activate the *VEGF* gene (Figure 1.). Activation of VEGF leads to increased angiogenesis to counteract hypoxia.^30^ Glioblastoma multiforme tumors are often hypoxic and have increased VEGF expression that contributes to the irregular vasculature associated with GBM. GBM tissues have been shown to have very high levels of VEGF expression that is associated with an up-regulation of the VGFR receptor VEGFR2.^31,32^

### PDGFR

PDGF/PDGFR signaling is involved in the development of normal tissues and its dysregulation contributes to oncogenesis. GBMs regularly exhibit a PDGF autocrine loop that is absent in normal brain tissues. This observation pinpoints to the importance of PDGF in GBM pathology.^33,34^ Data analyses from TCGA research network revealed amplification of platelet-derived growth factor receptor alpha (PDGFRα) in 10–13% of the cases studied.^9,10^ PDGFRα is the second most frequently amplified RTK in GBM behind EGFR. Glioblastoma multiforme has been shown to express all PDGF ligands (PDGF-A, PDGF-B, PDGF-C and PDGF-D) and the two cell surface receptors: PDGFR-α and PDGFR-β.^21^

### HGFR/c-MET

Scatter factor (SF)/hepatocyte growth factor (HGF) is the activating ligand for HGFR/c-MET that have been shown to be secreted by brain tumor cells. HGFR/c-MET expression and activation in tumor cells and vascular endothelial cells, results in cellular proliferation and invasion.^35^ The association of HGFR/c-MET with proliferation and survival indicates its suitability as a target for GBM therapy. HGFR/c-MET amplification was detected in 1.6–4% of human GBMs studied.^9,10^ Expression of HGFR/c-MET has been linked with poor prognosis for GBM patients.^36,37^

### FGFR

Humans have 22 FGFs (fibroblast growth factors) and four different FGF receptors (FGFR1, 2, 3 and 4).^38^ FGFR amplification was identified in 3.2% of the cases studied by TCGA.^10^ FGF2 has been shown to stimulate growth of cultured GBM cell lines and inhibition of FGFR signaling by RNA interference or by antibody blockade reduced GBM cell proliferation.^39^ FGFR1 has also been shown to be expressed at higher levels in brain tumors and when compared to adjacent normal brain tissue, suggesting a role for this receptor in tumorigenesis.^40,41^ FGF5 has also been shown to be overexpressed in GBM and this expression was linked to increased proliferation.^41^

### IGF-1R

GBM cell lines and tissues have been shown to express the IGF-1R.^42,43^ IGF-1R was seen to be overexpressed in GBM, and this overexpression was linked to shorter survival and reduced responsiveness to temozolomide, hinting at the role of IGF-1R signaling in GBM pathogenesis.^44^

## Targeting the tyrosine kinase receptors

### Small-molecule kinase inhibitors

Many molecules that target the kinase domains of RTKs have been tested in the context of GBM (Table 1). Erlotinib is an EGFR tyrosine kinase inhibitor that prevents the autophosphorylation of the tyrosine kinase intracellular domain of EGFR.^45^ It has been tested in several phase II studies for GBM and in conjunction with temozolomide for newly diagnosed GBM. The combination of the two drugs was well tolerated by patients and resulted in improved survival.^45^ However, treatment with Erlotinib alone was not effective in patients with recurrent GBM.^46^ Gefitinib (ZD1839/Iressa) is also an EGFR tyrosine kinase inhibitor that has been shown to radiosensitize U251 GBM cells *in vitro*.^47^ When tested at phase II trial, Gefitinib did not lead to an improvement in overall and progression free survivals for patients with newly diagnosed GBM.^48^

Multiple kinase inhibitors such as AEE788 and Vandetanib target both EGFR and VEGFR tyrosine kinases (Table 1). When tested in GBM patients, these drugs appeared to have little efficacy or increased toxicity. AEE788 was shown to have highly toxic side effects and very little efficacy for the treatment of recurrent GBM at phase I clinical trial,^49^ whereas Vandetanib had very little effect *in vitro* on GBM cell lines. However, when combined with histone deacetylase inhibitors (HDACIs), Vandetanib reduced GBM cell proliferation *in vitro*.^50^ The incorporation of Vandetanib to the standard therapy regimen (surgery+chemotherapy+radiotherapy) in phase II trial, also yielded little effect on overall survival and resulted in early termination of trial.^51^ Lapatinib is another multiple kinase inhibitor that binds both EGFR and HER2 tyrosine kinases and prevents their activation. In a phase I/II trial for recurrent GBM, it was shown to have little effect on patients.^52^ However, CUDC-101 a multi-targeted EGFR/HDAC (histone deacetylase) inhibitor has been shown to enhance the radiosensitivity of GBM cell lines *in vitro*.^53^

Vatalanib (PTK787), Sorafenib and Tivozanib are VEGFR tyrosine kinase inhibitors that have been found to have little efficacy on GBM patients when administered individually (Table 1). Vatalanib (PTK787) is well tolerated by patients but it does not appear to result in tumor regression.^54^ Likewise, the combination of Sorafenib with standard therapy also resulted in little effect on the treatment efficacy for GBM at the phase II stage.^55^ In a phase II study for patients with recurrent GBM, Tivozanib had apparent anti-angiogenic effects, but failed to affect tumor volume.^56^

Cediranib (AZD2171), a VEGFR-2 tyrosine kinase inhibitor, has been used as a monotherapy or in combination with Lomustine chemotherapy for recurrent GBM in phase III trial. Cediranib failed to improve progression free survival as a monotherapy and in conjunction with Lomustine.^57^ Similarly, Vandetanib a dual inhibitor of VEGFR-2 and EGFR that was tested in a phase II trial for recurrent GBM also had little efficacy in patients with GBM.^58^

The multiple kinase inhibitor Sunitinib is an inhibitor of VEGF, PDGFR, FLT1, FLT1/KDR, FLT3 and the RET kinases.^59^ In a phase II study for recurrent glioblastoma multiforme, Sunitinib was found to be unsuitable as a monotherapy with all patients’ disease progressing despite treatment.^60^

PDGFRα, PDGFRβ, Bcr-Abl, c-FMS and c-Kit tyrosine kinases can be targeted using the kinase inhibitor Imatinib (Gleevec/ST1571). This molecule disrupts the ligand-receptor autocrine loops for PDGFR.^61^ Likewise, this drug appeared to have little beneficial activity for GBM patients in phase II study.^62^ On the other hand, Tyrphostin (AG-1296), also a PDGFR-α, PDGFR-β, c-Kit, FMS-like tyrosine kinase 3 and a BEK tyrosine kinase inhibitor, was shown to reduce GBM cell viability *in vitro* and to have anti-tumor activity in a murine xenograft model of GBM.^63^ Tandutinib which targets PDGFR-β, FMS-like tyrosine kinase 3 and c-Kit, was tested in phase II trial in patients with recurrent GBM however this trial was halted due to the drug’s lack of efficacy.^64^ Other multi-kinase inhibitors such as Lenvatinib (E7080) and Nintedanib that inhibit VEGFR, FGFR and PDGFR kinases were tested in phase II studies. Although only Lenvatinib appeared to have modest activity on recurrent GBM patients, therapy with this inhibitor was accompanied with high toxicity in GBM treated patients.^65–67^

XL-184 (BMS-907351/Cabozantinib) is an oral inhibitor of c-MET, VEGFR-2 and RET,^68^ and it also has an inhibitory effect on KIT, FLT3 and TEK.^69^ Initial results from a phase II trial using XL-184 are promising, but further research is required to fully test its efficacy for GBM.^68^ Other molecules such as Foretinib and SGX-523 inhibit HGFR/c-MET tyrosine kinase and have been shown to reduce tumor growth *in vitro* and *in vivo* when using a GBM murine xenograft model.^70,71^ PD173074 is another multiple tyrosine kinase inhibitor that inhibits FGFR and VEGFR tyrosine kinases. PD173074 showed GBM growth inhibitory effects *in vitro*^39^ and as a result this drug might be of benefit for GBM patients. PQ401, GSK1838705A, PPP (picropodophyllin/AXL1717) and NVP-AEW541 are IGF-1R tyrosine kinase inhibitors that have all shown promising results pre-clinically (Table 1). PQ401 has been shown to suppress GBM cell growth and migration *in vitro*.^72^ GSK1838705A induced apoptosis of GBM cells *in vitro,* and when these cells were implanted in nude mice GSK1838705A had similar anti-GBM activity.^73^ PPP (Picropodophyllin/AXL1717) was shown to inhibit the growth of GBM cell lines that led to *in vivo* regression of intracranial xenografts.^74^ NVP-AEW541 induces apoptosis in GBM cell lines *in vitro* when co-administered with Dasatinib (a Bcr-Abl tyrosine kinase inhibitor).^75^ BMS-536924 is an ATP competitive IGF-1R/IR (insulin receptor) inhibitor that has shown promising anti-tumor properties *in vitro* and when tested on Temozolomide (TMZ) resistant GBM cells.^76^

These small-molecule inhibitors have been widely studied in many cancers, with varying degrees of success, however the clinical trial data for GBM shows that very few of these molecules have a significant anti-tumor response, and thus other components of the RTK receptors are being considered as therapeutic targets.

### Antibody therapies targeted at RTKs’ extracellular domain

Whilst many therapies target the kinase domain of RTKs, the extracellular domain is also a viable target when using antibody therapies. These molecules are being used as antagonists of the ligand-binding domains of RTKs with the aim of preventing ligand-binding and subsequent activation of the kinase domains. A monoclonal EGFR targeting antibody known as Cetuximab has been utilized as a therapy for GBM. This antibody targets the extracellular domain of EGFR, and acts as an antagonist that prevents the activation of RTKs and therefore, inhibits tumor malignancy.^77^ Cetuximab has been tested as a salvage therapy for patients who have failed to respond to surgery, radiation therapy and chemotherapy. Although this monotherapy proved to be well tolerated, its activity for recurrent glioblastoma multiforme was minimal at phase II clinical trial.^78^ Other antibodies such as Ornartuzumab have been used to target the extracellular domain of the HGFR/c-MET receptor and this has been shown to inhibit orthotopic U87 GBM xenograft tumor growth.^79^ MK-0646 (H7C10/F50035/Dalotuzumab) a humanized monoclonal IGF-1R antibody that acts as an antagonist, has also been shown to reduce cell proliferation and to induce apoptosis.^80^ Although these antibody therapies are still in their relative infancy compared to the small-molecule inhibitors of the RTK kinases, early research has been promising in the context of GBM. It is important to note that due to their large size antibodies do not freely cross the blood–brain barrier, thus there is a need to engineer antibodies to enable them to cross the blood–brain barrier and access GBM tumors. Only around 0.1–0.2% of circulating antibodies have been shown to penetrate the blood–brain barrier.^81^ Bispecific antibodies consist of two different single chain Fv fragments connected by a linker. Directed antibodies with optimized binding to the transferrin receptor have been used to cross the blood–brain barrier in both murine and primate models.^82^ These engineered antibodies are exciting new therapeutics that enable the crossing of the blood–brain barrier and direct targeting of tumor cells. Alternatively antibodies can also be delivered directly into the brain using Ommaya reservoirs or at the time of surgery to bypass the blood–brain barrier.

### Therapies directed at RTK ligands

Antibodies have also been used to ‘trap’ the ligands that activate RTK signaling pathways. Bevacizumab is a humanized murine monoclonal antibody that binds VEGF and prevents its binding to the receptor. This antibody was granted accelerated approval by the FDA (food and drug administration) in 2009 for the treatment of patients with progressive or recurrent GBM. Despite its approval, Bevacizumab has been shown to have little efficacy for newly diagnosed GBM. The addition of Bevacizumab to the current course of therapy conveys no benefit for overall patient survival.^83^ Aflibercept is another VEGF ‘trap’ that binds VEGF and prevents its interaction with the receptor. In phase II trial Aflibercept appeared to have little activity for recurrent GBM patients with only 7.7% of patients experiencing progression free survival after 6 months.^84^ Rilotumumab (AMG102) is an anti-HGF monoclonal antibody that binds HGF and prevents its binding to the HGFR/c-MET, and consequent activation of downstream targets. When combined with temozolomide *in vitro*, Rilotumumab has been proven to inhibit the growth of U87MG glioblastoma multiforme cells.^85^ In a phase II clinical study, this antibody showed little effect for the treatment of recurrent glioblastoma multiforme.^86^ Although targeting these ligands is an attractive avenue for GBM therapy, the efficacy of these therapies has been limited. This may be due to factors such as RTK receptors being mutated and constitutively active, such as mutations encountered in EGFRvIII. The blood–brain barrier may also present an issue for these antibody therapies, preventing their tumor penetration, inhibiting their anti-tumor effects.

## RTK downstream signaling pathways

### The PI3K/AKT/mTOR pathway

The PI3K/AKT/mTOR pathway is activated by transmembrane tyrosine kinase growth factor receptors, transmembrane integrins and G-protein-coupled receptors (Figure 1). Upon activation of these receptors, functional PI3K translocates to the plasma membrane and leads to the production of phosphatidylinositol 3,4,5-triphosphate (PIP_3_) from phosphatidylinositol bisphosphate (PIP_2_).^87,88^ PIP_3_ activates serine/threonine kinase phosphoinositide-dependent kinase 1 (PDK1) and AKT (at threonine 308).^87,88^ Phosphatase and tensin homolog (PTEN) acts to counteract PI3K signaling by dephosphorylating PIP_3_ to PIP_2_.^89^ Activated Akt phosphorylates the FOXO subfamily, which inhibits the transcription of several pro-apoptotic proteins, it can also inhibit apoptosis by phosphorylating and inactivating pro-apoptotic proteins such as BAD and GSK3.^88,90^ Other functions include the phosphorylation and degradation of the inhibitor of κB (IκB), and which results in increased nuclear factor kappa B (NF-κβ) activity and transcriptional stimulation of pro-survival genes,^91^ it also modulates MDM2, which inhibits P53 (an activator of cell-cycle arrest).^92^

Akt directly and indirectly leads to activation of mTOR which is present in two distinct complexes: mTORC1 and mTORC2. mTORC1 is composed of mTOR, Raptor, mLST8 and PRAS40. mTORC1 activates S6K1 and subsequently S6, resulting in increased cell proliferation and growth. It also leads to the inhibition of eIF4E binding protein 1 (4E-BP1), which allows the formation of eukaryotic initiation factor 4F (eIF4F) and protein translation.^93^ mTORC2 is composed of mTOR, Rictor, Sin1 and mLST8 and its role is less understood.^93^ It has been found that mTORC2 activates PKC, promoting its kinase activity.^94^ It is also thought that mTORC2 may take part in cell survival and cytoskeletal organization.^95^ mTOR has been shown to regulate hypoxia-inducible factor 1α (HIF1α), leading to downstream activation of vascular endothelial growth factor (VEGF) secretion and increased angiogenesis.^96^

### The Ras/MAP/ERK pathway

This signaling pathway is activated by cell surface receptors and regulates the activity of many cellular factors involved in angiogenesis, cell proliferation, migration and survival (Figure 1). The activation of Ras protein by the exchange of GDP with GTP, results in the activation of MAP kinases that also activate downstream ERK via phosphorylation.^97^ This pathway is often activated in certain tumors by mutations in cytokine receptors such as Flt-3, Kit, Fms or by overexpression of wild-type or mutated receptors.^98^ Activation of the Ras/MAP/ERK pathway also leads to activation of HIF-1α, which promotes tumorigenesis and activation of VEGF.^99^

### RTK signaling pathways in GBM pathogenesis

A large percentage of mutations and deletions in the RTK signaling pathways are evident in numerous cancers including GBM. The RTK/Ras/PI(3)K pathway was found to be altered in 86–90% of GBM cases studied.^9,10^ Combined activation of the Ras and AKT pathways has been shown to induce glioblastoma tumor formation in mice.^100^ The AKT signaling pathway plays a pivotal role in the progression of grade III anaplastic astrocytoma to grade IV glioblastoma multiforme. AKT expressing tumors appear to grow at a faster rate than non-AKT expressing tumors.^101^ Furthermore, inhibition of the PI3K/AKT pathway has been shown to inhibit the growth of GBM cells,^102^ further highlighting the importance of this pathway in GBM pathogenesis.

Inhibitors of the PI3K/AKT/mTOR signaling pathway are also affected in GBM. As an example, PTEN is mutated or deleted in approximately 36–44% of GBM cases.^9,10,103^ Loss of PTEN function has also been linked to immune evasion seen in GBM tumors, with mutations of PTEN being linked to increased expression of the immune suppressive checkpoint PD-L1.^104^ Another example is the tumor suppressor Neurofibromin 1 (NF1) that inhibits Ras.^105^ NF1 has a region that is highly homologous to the catalytic domain of Ras GTPase-activating protein (p120GAP), and consequently, it stimulates Ras GTPase, which leads to Ras bound GTP hydrolysis into GDP, and the inactivation of Ras activity.^106^ NF-1 is involved in the development of GBM as evidenced by the correlation between neurofibromatosis type-1 (a disease characterized by NF-1 mutation) and GBM occurrence.^107^

## Targeting RTK signaling pathways in GBM

### PI3K

Although therapies targeting PI3K in GBM have shown promising results *in vitro* and *in vivo* using xenograft models, their clinical efficacy remain to be tested and/or proven. PX-866 (Sonolisib) is an irreversible PI3K inhibiting drug that has been shown to inhibit angiogenesis and invasion of GBM cells *in vitro.* Although the drug did not induce apoptosis of GBM cells, it did cause cell cycle arrest.^108^ This drug was tested in a phase II trial for recurrent glioblastoma and was well tolerated but 73% of patients treated had disease progression.^109^ Other inhibitors such as XL765 (SAR245409) and GDC-0084, dual PI3K/mTOR inhibitors, have anti-GBM effects *in vitro* and *in vivo* but their efficacy in clinical trials must be tested and presented.^110,111^

### mTOR

Several mTOR inhibitors have been trialed for GBM with differing results. As an example, Temsirolimus (CCI-779), Sirolimus (Rapamycin) and Everolimus (RAD001) are mTOR inhibitors that were shown to have little efficacy on GBM treatment. Temsirolimus failed to show efficacy for recurrent GBM in Phase II clinical trial.^112^ Sirolimus also had little efficacy for treatment of recurrent GBM patients even when combined with the EGFR tyrosine kinase inhibitor Erlotinib.^113^ Similarly, Everolimus did not convey a significant survival benefit when combined with temozolomide and radiotherapy in a phase II trial for newly diagnosed GBM patients.^114^ On the other hand, AZD2014 (Vistusertib), CC-223 (TORKi) and Palomid 529, which are dual mTORC1/mTORC2 inhibitors, have shown therapeutic promise. AZD2014 (Vistusertib) radiosensitized glioblastoma stem-like cells *in vitro* and *in vivo*.^115^ As a result of these promising preclinical results, participants are being recruited for a phase I/II clinical trial and from previously treated GBM patients (clinical trial ID: NCT02619864). CC-223 (TORKi) was found to exhibit anti-tumor effects in a murine xenograft model of GBM (utilizing U87MG cells)^116^ and Palomid 529 hindered GBM tumor growth in an orthotopic murine tumor model.^117^

## RAS targeting by aminobisphosphonates: nanotech-based strategies

Ras is another valid therapeutic target for the treatment of GBM. Aminobisphosphonates are promising anti-cancer therapeutics, these drugs are thought to disrupt cancer proliferation, invasion, survival and pro-angiogenic activity by inhibiting the synthesis of farnesyl and geranyl lipidic residues, which in turn prevents protein isoprenylation. Ras is a farnesylated protein that it is inhibited by aminobisphosphonates, this inhibition prevents Ras GTPase activity and prevents downstream signaling.^118^ Zoledronic acid (ZOL) is an aminobisphosphonate that has anti-cancer effects, however it is mainly used to treat bone metastases as it accumulates in the bone, as a result novel methods are required to deliver this drug extra-skeletally.^119^ Nanotechnology can be utilized to help prevent bone accumulation of ZOL and ensure blood–brain barrier penetration of the drug. Salzano G *et al.* developed self-assembling nanoparticles that target transferrin receptors via incorporation of transferrin known as Tf-PLCaPZ. Tf-PLCaPZ encapsulates zolderonic acid and delivers it across the blood–brain barrier. Tf-PLCaPZ showed significant *in vitro* LN229 cell growth inhibition, Tf-PLCaPZ also showed anti-tumor activity *in vivo* in a U373MG xenograft model.^120,121^ These promising preclinical results make ZOL an exciting potential therapy for GBM.

## The RAF serine/threonine kinase

Raf is a component of the Ras/Raf/MEK/ERK signaling pathway that can be targeted for GBM treatment (Figure 1). Sorafenib, a Raf kinase inhibitor, has been tested in combination with Erlotinib (an EGFR tyrosine kinase inhibitor) and in a phase II trial for patients with recurrent GBM (Table 2). This combinational therapy did not appear to have the desired beneficial effects, as it failed to reach the goal of a 30% improved survival time. It was postulated that this may be due to pharmacokinetic interaction between the drugs which reduces their efficacy.^122^

## Protein kinase C (PKC)

Protein kinase C (PKC) family members regulate several cellular responses including gene expression, protein secretion, cell proliferation, and the inflammatory response. Tamoxifen is an inhibitor of PKC that has been tested as a therapeutic compound for GBM. In a phase I study, Tamoxifen was well tolerated but when combined with radiotherapy it did not appear to radiosensitize GBM tumors, as was observed *in vitro*.^123^ In a phase II trial combining high-dose Tamoxifen and radiotherapy, it was found that this molecule did not increase survival of patients.^124^ Worryingly, it was reported that high-dose tamoxifen treatment was linked with multifocal glioblastoma recurrence which mainly occurred in patients who responded to the Tamoxifen treatment.^125^ Enzastaurin is an inhibitor of the PKCβ and PI3K/AKT pathways that has been tested in phase I and phase II clinical trials. When compared with the alkylating chemotherapeutic drug Lomustine in a phase III trial for recurrent GBM, Enzastaurin did not display better efficacy.^126^

## The tumor suppressor p53

**Figure 2:**
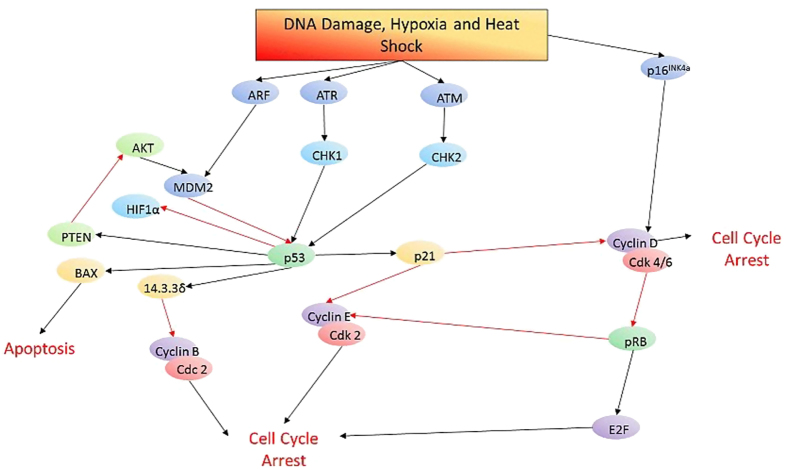
A schematic representation of the role of p53 and pRB signaling in response to stress signals. Black arrows indicate activation whereas red arrows indicate inhibition.

The p53 pathway is altered in a large variety of cancers, with GBM being no exception. 87% of cases studied by the Cancer Genome Atlas Research Network had alteration of the p53 signaling pathway, with p53 being mutated or deleted in 28–35% of cases.^9,10^ The p53 protein pathway is involved in the activation of genes that are implicated in cell cycle arrest and apoptosis (Figure 2).^127^ Stress signals, such as DNA damage, hypoxia, heat shock and cold shock elicit a p53 response. These stress signals also result in the activation of mouse double minute 2 homolog (MDM2), a protein that degrades p53.^128^ The p53 protein activates p21 that inhibits Cdk4/Cyclin D and Cdk2/Cyclin E complexes and prevents their cell cycle progression.^129^ Upon p53 activation the transcription of Cyclin B is also reduced, preventing cell cycle progression.^130^ Due to the importance of p53 in GBM pathogenesis, a gene therapy approach has been used to restore p53 expression. SGT-53 is a nanocomplex that delivers wild-type p53 to tumor cells. It was shown to sensitize Temozolomide resistant tumor cells to treatment *in vitro* and *in vivo*.^131^ Introduction of wild-type p53 into Temozolomide resistant GBM cells resulted in a reduction of MGMT protein expression and this may explain the improved responsiveness to TMZ observed.^131^ In a phase I trial, intratumoral delivery of wild-type p53 gene using an adenovirus (Ad-p53) caused apoptosis of transfected tumor cells, indicating a beneficial anti-tumor effect.^132^

## The tumor suppressor pRb

The pRB pathway suppresses cell cycle entry and progression via its interaction with the transcription factor E2F, leading to down regulation of genes involved in cell cycle progression.^133–135^ The pRB pathway was altered in 78–79% of GBM cases studied with *RB* gene deletion or mutation in 7.6–11% of cases.^9,10^ As a result therapies have been developed to reactivate pRb. PD0332991 (Palbocilib) is an inhibitor of Cdk4/6, that prevents the downstream inhibition of pRb (Figure 2). PD0332991 (Palbocilib) has been shown to inhibit the growth of intracranial GBM xenograft tumors.^136^

## *O*^6^-methylguanine-DNA methyltransferase (MGMT)

MGMT is an enzyme that conveys a resistance to temozolomide chemotherapy (the standard chemotherapy of choice for GBM. MGMT acts alone to remove the methyl lesions caused by temozolomide.^137^ A single MGMT molecule removes the *O*^6^-methylation on guanine in a single step and transfers the methyl group from the oxygen in the DNA to a cysteine residue in the active site of MGMT.^137^ The binding of the methyl group irreversibly inactivates MGMT. Once the methyl group is bound MGMT is ubiquitinated and degraded by the proteasome.^138^ The repair of *O*^6^-methylation on guanine is biphasic with an initial fast repair phase followed by a slower phase caused by the depletion and subsequent synthesis of MGMT.^138^ *O*^6^-benzylguanine blocks the active site of MGMT, inactivating it and allowing methyl adducts to accumulate. In a preclinical model using xenotransplanted nude mice, the combination of *O*^6^-benzylguanine with temozolomide or carmustine (BCNU), amplified the anti-tumor effects of these chemotherapeutic agents.^139^ These promising preclinical results were unfortunately not replicated at the clinical trial phase. *O*^6^-benzylguanine did not re-sensitize temozolomide resistant patients to temozolomide chemotherapy as expected and as a result the drug was not investigated past the phase II trial stage.^140,141^

## TGF-β signaling

TGF-β is a cytokine that exerts its effects on many cell types and is involved in the regulation of cell growth, immunity, cell death and cell adhesion.^142^ TGF-β binds to TGF-β receptor II (TGF-ΒRII) resulting in the formation of a heterodimer with the TGF-β receptor I (TGF-ΒRI) and leading to the phosphorylation of TGF-ΒRI.^143^ This event results in TGF-ΒRI the phosphorylation and activation of SMAD proteins. Once activated, the SMADs form complexes that in turn regulate the expression of target genes,^143^ it is important to note that TGF-β also signals via non-SMAD pathways.^144^ TGF-β signaling has been shown to facilitate Ras/Raf/MEK/ERK signaling via the increased GTP loading of Ras. TGF-β has also been shown to activate the PI3K/AKT/mTOR pathway.^145^ In healthy conditions TGF-β acts as a tumor suppressor, inhibiting proliferation, as a result mutations in the TGF-β signaling pathway, lead to an insensitivity to this cell growth prevention.^143^ Aberrant TGF-β signaling results in inflammation, invasion, metastasis, angiogenesis and immune escape. In GBM the TGF-β pathway is dysregulated and contributes to pathogenesis and progression.^143^ GBM cells have been shown to secrete TGF-β2 that also suppresses the anti-GBM immune response.^146^

AP12009 (Trabedersen) is a TGF-β2-specific antisense oligonucleotide, that when delivered using convection enhanced delivery (CED) resulted in a longer median overall survival (in phase I/II trial).^147^ SB-431542, LY2109761 and LY364947 (HTS466284) are inhibitors of the TGF-βR1 tyrosine kinase that have been tested in the GBM setting. SB-431542 has been shown to inhibit GBM cell growth, and motility *in vitro*.^148^ LY2109761 delivery in conjunction with radiotherapy improves GBM tumor responsiveness to radiotherapy in an orthotopic murine model.^149^ LY2109761 also has been shown to delay tumor growth in murine xenografts when used as a monotherapy and when combined with TMZ chemotherapy.^149^ LY364947 (HTS466284) has also been shown to increase the sensitivity of GBM cells to radiotherapy.^150^

## Conclusions

Glioblastoma multiforme is an elusive disease with a dismal prognosis, and alternative therapies are required to improve the prognosis for patients. Genomic analyses of GBM uncovered several dysregulations of key cellular signaling pathways that constitute attractive targets for therapy. Targeting individual components of these pathways using small-molecule inhibitors and antibodies has provided varying levels of success in the treatment of GBM. Therefore, it may be more advantageous to target multiple elements of various signaling pathways, to eradicate GBM. It is also important to note that tumor cells are heterogeneous, and a targeting strategy that is aimed at multiple pathways would constitute a more efficient therapy. Many therapies also fail to have beneficial effects due to the blood–brain barrier and the presence of active efflux pumps that prevent drug entry into the brain. One such example of receptor tyrosine kinase inhibitors that have low brain penetration rates are Erlotinib and Gefitinib which have cerebrospinal fluid penetration rates as low as 2.8–4.4% and 1.1–1.3% respectively.^151^ The drug transporters P-glycoprotein (P-gp) and breast cancer resistance protein (BCRP) have been shown to reduce brain penetration of Erlotinib explaining the relatively poor results seen in the GBM setting.^152^ Recent advances in nanoparticle delivery of drugs have enabled the delivery of drugs previously incapable of crossing the blood–brain barrier, reach the brain parenchyma and thus, enable effective targeting of intracranial tumors.^153,154^ The combination of focused ultrasound with microbubbles has also been shown to allow drugs to penetrate the blood–brain barrier.^155–157^ This technique may allow RTK inhibitors to cross the blood–brain barrier more efficiently and therefore enhance their effects. Immunotherapy may also be used as an alternative therapy with targeted immune cells crossing the blood–brain barrier. Numerous promising immunotherapies using peptide-targeted vaccines are entering clinical trials and preliminary results are proving to be beneficial for patients.^158,159^ Dendritic cell vaccines also showed encouraging results at the clinical trial stage.^160^ With these novel therapies comes hope for the future treatment of GBM.

## Figures and Tables

**Table 1:** Examples of drugs that target the intracellular components of the RTK pathways that have undergone testing in GBM

*Tyrosine kinase receptor*	*Target*	*Drug*	*Preclinical anti-tumour activity*	*Clinical trials*	*References*
EGFR	EGFR	Erlotinib (Tarceva)	Activity seen *in vitro* and on *in vivo* xenografts	Phase II for newly diagnosed and recurrent GBM	45,46
		Gefitinib (ZD1839/Iressa)	Radiosenitisation observed *in vitro*	Phase II for newly diagnosed GBM	47,48
	EGFR and VEGFR	AEE788 (Everolimus)	Activity seen *in vitro* and on *in vivo* Xenografts	Phase I for recurrent GBM	49
		Vandetanib (ZD6474)	Activity seen *in vitro* when combined with HDACs	Phase II for newly diagnosed GBM	50,51
	EGFR and HER2	Lapatinib	—	Phase I/II for recurrent GBM, Phase II trial for newly diagnosed GBM currently recruiting NCT01591577	52
	EGFR/HDAC	CUDC-101	Radiosensitisation observed *in vitro*	—	53
	EGFR extracellular domain	Cetuximab (Erbitux)	Radiosensitisation observed *in vitro*	Phase II for recurrent GBM, Phase I/II trial for newly diagnosed GBM currently recruiting NCT02861898, Phase II trial for relapsed/refractory GBM recruiting NCT02800486, Phase I/II trial for relapsed/refractory GBM + Bevacizumab NCT01884740	77,78
VEGFR	VEGF-1	Bevacizumab	—	Phase III for newly diagnosed GBM	83
	VEGFR	Vatalanib (PTK787)	*In vitro* activity	Phase I for newly diagnosed GBM	54
		Sorafenib	—	Phase II for newly diagnosed GBM, Phase II in combination with Everolimus currently recruiting NCT01434602	55
		Tivozanib	—	Phase II for recurrent GBM	56
	VEGFR-2	Cediranib (AZD2171)	—	Phase III for recurrent GBM in combination with Lomustine	57
	VEGFR-2 and EGFR	Vandetanib	Activity seen on *in vivo* xenografts	Phase I/II for recurrent GBM	58
	VEGFR, PDGFR, FLT1, FLT1/KDR, FLT3 and the RET kinases	Sunitinib	*In vitro* and *in vivo* activity observed	Phase II for recurrent GBM, Phase II trial for newly diagnosed GBM patients recruiting NCT02928575	60
	PGFR, FGFR and VEGFR	Nintedanib (BIBF1120)	—	Phase II for recurrent GBM	65,66
	VEGF	Aflibercept	*In vitro* and *in vivo* activity observed on U87MG xenografts	Phase II for recurrent GBM	84
PDGFR	PDGFRα, PDGFRβ, Bcr-Abl, c-FMS and c-Kit	Imatinib mesylate (Gleevec/ST1571)	—	Phase I/II for recurrent GBM	62
	PDGFRα, PDGFRβ, c-Kit, FMS-like tyrosine kinase 3 and BEK	Tyrphostin	Reduced cell viability *in vitro* and *in vivo*	—	63
	PDGFRβ, FMS-like tyrosine kinase 3 and c-KIT	Tandutinib	—	Phase I/II for recurrent GBM	64
	VEGFR, FGFR and PDGFR	Lenvatinib (E7080)	—	Phase II for recurrent GBM	67
		Nintedanib (BIBF1120)	—	Phase II for recurrent GBM	65,66
HGFR/c-MET	c-MET, VEGFR-2, RET, KIT, FLT3 and TEK	XL-184 (BMS-907351/Cabozantinib)	—	Phase II in previously treated GBM patients	68
	c-MET and VEGFR-2	Foretinib	*In* vitro and *in vivo* activity observed	—	70
	c-MET	SGX-523	*In* vitro and *in vivo* activity observed	—	71
	HGF	Rilotumumab (AMG102)	*In vitro* activity observed	Phase II for recurrent GBM	85,86
	c-MET extracellular domain	Ornartuzumab	Reduces xenograft growth *in vivo*	Phase II for recurrent GBM	79
FGFR	FGFR, PDGFR and VEGFR	Nintedanib (BIBF1120)	—	Phase II for recurrent GBM	65,66
		Lenvatinib (E7080)	—	Phase II for recurrent GBM	67
	FGFR, VEGFR	PD173074	*In vitro* growth inhibition	—	39
IGF-1R	IGF-1R	PQ401	*In vitro* activity	—	72
		GSK1838705A	*In vitro* and *in vivo* activity	—	73
		PPP (Picropodophyllin/AXL1717)	*In vitro* and *in vivo* intracranial activity	Phase I/II trial for recurrent GBM currently recruiting NCT01721577	74
		NVP-AEW541	*In vitro* activity observed	—	75
	IGF-1R/IR	BMS-536924	*In vitro* and *in vivo* activity observed	—	76
	IGF-1R extracellular domain	MK-0646 (H7C10/F50035/Dalotuzumab)	*In vitro* activity observed	—	80

**Table 2:** Examples of drugs that target the tyrosine kinase receptors that have been tested in GBM

*Target*	*Drug*	*Preclinical activity*	*Clinical trials*	*References*
PI3K	PX-866	*In vitro* cell cycle arrest and *in vivo* tumour growth inhibition	Phase II for recurrent GBM	108,109
mTOR	Temsirolimus (CCI-779)	*In vivo* activity on cells implanted in nude mice	Phase II for recurrent GBM	112
	Sirolimus (Rapamycin)	—	Phase II for recurrent GBM in combination with Erlotinib	113
	Everolimus (RAD001)	—	Phase II for recurrent GBM in combination with Sirolimus, Phase II for newly diagnosed GBM, Phase I/II trial with Sorafenib for recurrent GBM currently recruiting NCT01434602	113,114
	AZD2014 (Vistusertib)	Radiosensitisation of GBM stem-like cells *in vitro*	Phase I/II for previously treated GBM currently recruiting NCT02619864	115
	CC-223 (TORKi)	*In vivo* Xenograft activity	—	116
	Palomid 529	*In vivo* orthotopic xenograft GBM activity	—	117
PI3K/mTOR	XL765	*In vitro* and *In vivo* activity	—	110
	GDC-0084	*In vitro* and *In vivo* activity observed	Phase I trial for recurrent GBM has been completed NCT01547546	111
PKCβ/PI3K	Enzastaurin	*In vitro* and *in vivo* activity observed using GBM cell lines and murine xenografts	Phase III for recurrent GBM	126
Ras	Zoledronic acid	*In vitro* and *in vivo* activity	—	120,121
RAF	Sorafenib	—	Phase II trial in combination with Erlotinib for recurrent GBM, Phase I/II trial with Everolimus for recurrent GBM currently recruiting NCT01434602	122
PKC	Tamoxifen	—	Phase II for newly diagnosed GBM	123–125
p53	SGT-53	*In vitro* chemosensitisation of GBM cell lines to TMZ and *In vivo* activity when combined with TMZ	Phase II study for recurrent GBM currently recruiting NCT02340156	131
	Ad-p53	—	Phase I trial for recurrent GBM	132
Cdk4/6	PD0332991 (Palbociclib)	*In vitro* GBM cell cycle arrest and *In vivo* intracranial xenograft growth inhibition	Phase I trial recruiting for young patients with nervous system tumours NCT02255461	136
